# Cervical Cytology Specimen Stability in Surepath Preservative and Analytical Sensitivity for HPV Testing with the cobas and Hybrid Capture 2 Tests

**DOI:** 10.1371/journal.pone.0149611

**Published:** 2016-02-23

**Authors:** Keith D. Tardif, Michael T. Pyne, Elisabeth Malmberg, Tatum C. Lunt, Robert Schlaberg

**Affiliations:** 1 ARUP Institute for Clinical and Experimental Pathology, Salt Lake City, UT, United States of America; 2 University of Utah School of Medicine, Department of Pathology, Salt Lake City, UT, United States of America; Georgetown University, UNITED STATES

## Abstract

None of the commercial HPV tests are U.S. FDA-approved for testing of cervical cytology specimens in SurePath preservative. Still, ~30% of HPV testing is performed on specimens in this formalin-containing preservative. Formalin-induced DNA fragmentation and cross-linking may interfere with HPV detection. We evaluated analytical sensitivity and specimen stability of the **cobas** 4800 HPV (Roche) and Hybrid Capture 2 HPV (HC2, Qiagen) tests with residual cervical cytology samples in SurePath preservative available within 1 week of collection. **Cobas** testing was performed with and without heating samples at 120°C for 20 min diluted 1:1 in an alkaline environment (pretreatment) to revert DNA crosslinking. Stability was tested after 2 weeks of storage at ambient temperature followed by ≤10 weeks at 4°C. Analytical sensitivity and positivity rates (HC2, 18%; **cobas** pretreated, 46%; **cobas** untreated, 47%) were greater for **cobas** than HC2 (*n* = 682). After 6 weeks of storage, mean HC2 ratios were lower (mean 0.9, SD 6.3) but high variability limited statistical power to detect trends. **Cobas** threshold cycles (Ct’s) increased in untreated (mean 2.1) but not pretreated samples (mean 0.3; *n* = 110; *p≤*0.0001). Overall, **cobas** had greater analytical sensitivity for samples in SurePath preservative. Although pretreatment introduced a manual sample transfer step and 30 min of incubation times, it improved stability without negatively affecting analytical sensitivity. While awaiting results of large trials to evaluate the clinical performance of **cobas**, the addition of the pretreatment step may improve the detection of HPV, especially after prolonged sample storage.

## Introduction

Persistent infections with oncogenic, or high-risk (HR), human papillomavirus (HPV) genotypes can cause cervical cancer [1]. Current cervical cancer screening guidelines in the United States employ combinations of cervical cytology and HPV testing [2]. FDA-cleared HPV tests have high clinical sensitivity resulting in high negative predictive value, which has been used to extend recommended screening intervals [3–8]. Most cervical cytology specimens are collected in liquid based cytology (LBC) media, PreservCyt or SurePath preservatives. This allows the same LBC specimen to be used for cytology and HPV testing. In the U.S., approximately 30% of testing is performed with cervical samples collected in SurePath [9]. However, none of the commercial HPV tests are FDA-cleared for use with this preservative. Unlike PreservCyt, SurePath contains formalin, which crosslinks HPV DNA to proteins. DNA-protein cross-linking can result in DNA fragmentation and interfere with PCR amplification, thus reducing a test’s analytical sensitivity. Concerns of limited stability and reduced sensitivity of HPV testing using samples stored in SurePath preservative have been raised potentially causing false-negative results [10–15].

DNA-protein cross-linking can be reversed by heat treatment in the presence of a nucleophilic buffer (pre-analytical treatments) [16–18]. In a previous stability study, pretreated SurePath specimens were found to be stable for HPV testing with the **cobas** HPV Test for 10 weeks when stored at ambient temperature [19]. Untreated SurePath specimens were found to be stable at refrigerated temperatures for the same time period. However, results were only compared qualitatively and only a single time point. In addition, pooled HPV-positive specimens from a colposcopy referral population were used, which are expected to have higher concentration of HPV and are thus less likely to test negative after extended storage. Determining the stability of cervical samples in SurePath preservative and the need for a pretreatment step for HPV testing are critical for diagnostic laboratories as this has important implications on testing workflow.

In the present study, we serially monitored stability of cervical cytology samples in SurePath preservative with two commercial HPV tests (**cobas** HPV Tests and Digene Hybrid Capture 2, HC2) for up to 12 weeks with and without a pretreatment step using residual and pelleted patient samples. HC2 is FDA-cleared for HPV testing of cervical cytology samples in PreservCyte but not SurePath preservative. It uses RNA probes complementary to the entire ~8,000 base pair HPV DNA genome followed by detection of DNA-RNA hybrids by an antibody-mediated colorigenic reaction. The **cobas** 4800 HPV Test (Roche) is based on real-time PCR amplification of a ~200 nucleotide portion of the L1 region of the HPV genome. It is FDA-cleared for use with samples stored in PreservCyt but not SurePath preservative. Specimen stability was monitored using samples pooled within 7 days of collection. Pooled HPV positive-specimens were chosen for stability testing based on a range of **cobas** and HC2 results determined in experiments measuring test sensitivity and reproducibility.

## Materials and Methods

### Sample collection, preparation, and testing

Research performed on samples from human subjects was conducted following a protocol (IRB# 7275) approved by the University of Utah Institutional Review Board (IRB). No patient consent was required by the IRB because all samples used for this study were de-identified. Randomly selected cervical cytology samples in SurePath preservative were pooled within 7 days of collection. Pooling was necessary to ensure sufficient sample volume for testing of aliquots in sensitivity, reproducibility, and stability studies. Pools were tested with the **cobas** and HC2 tests immediately after pooling to limit the time between sample collections and testing. SurePath specimens were processed for HC2 testing by standard methods, including heat treatment at 65°C for 90 min. From each SurePath specimen (10 ml total volume) 8 mL were removed as part of routine cytology testing leaving at most 2 mL of residual sample for this study. For sensitivity experiments, 5 residual samples were pooled and the final volume was brought to 10 mL with fresh SurePath media if necessary. Because additional sample volume was needed for reproducibility and stability studies, 7 samples were pooled for these experiments and fresh SurePath media was added for a final volume of 14 mL if necessary. Positive and negative pools were identified by testing in the **cobas** (Roche Diagnostics, Indianapolis, IN) and HC2 tests (Qiagen, Gaithersburg, MD). Pools with discordant **cobas** and HC2 results were excluded from sensitivity and stability studies.

To simulate testing of sample that had been processed for cytology (pelleted samples), pooled SurePath samples were processed per standard protocol [20]. Briefly, 8 mL of residual SurePath specimen was layered onto 4 mL of BD PrepStain Density Reagent (Becton, Dickinson, and Company, Franklin Lakes, NJ) using the BD PrepMate. The layered samples were centrifuged at 200xg for 2 min. The supernatant was aspirated before the remaining fluid was centrifuged again at 800xg for 10 min. The supernatant was carefully removed by decanting. The pellet was resuspended in 1 mL of Tris buffered water and 2 mL of fresh SurePath media resulting in ~4 mL of final sample.

### Sensitivity

#### No pre-analytical treatment

HPV-positive pools used for sensitivity studies were ≤1 week old and HPV-negative pools were ≤2 week old.

**Residual and pelleted sample tested on the cobas and HC2:** For sensitivity studies comparing untreated residual and pelleted samples tested by **cobas** to residual and pelleted samples tested by HC2, 5 mL of HPV-positive pool was added to 10 mL of HPV-negative ‘master pool’. Master pools were generated by combining several HPV-negative pools to provide sufficient HPV-negative sample matrix for all serial dilutions in an experiment. Fourteen additional 3-fold serial dilutions were prepared. Aliquots from each dilution were tested on the **cobas** (1 mL) and HC2 (2 mL) tests in triplicate. For **cobas**, extracted DNA from each replicate was tested in duplicate. **Residual or pelleted sample tested on cobas vs. pelleted sample tested on HC2:** For sensitivity comparisons of pelleted HC2 specimen with pelleted or residual **cobas** specimen, **cobas** residual samples (1 mL) were tested in duplicate and 8 mL was used for pelleting from each 10 mL serial dilution. The pelleted sample from each dilution was tested on the **cobas** (1 mL) and HC2 (2 mL) tests with only one replicate due to limitations in sample volume.

#### Pre-analytical step

Effects of pre-analytical treatment on **cobas** test sensitivity were evaluated using sample pools screened with the **cobas** test with pre-treatment to maximize available sample volumes. For sample pretreatment, 0.5 mL of residual SurePath sample was mixed with 0.5 mL of pre-analytic buffer (190 mM Tris, 0.4% SDS, and 0.09 sodium azide, pH 8.5). This mix was then incubated at 120°C for 20 min, and after a 10 min cooling period, pretreated samples were analyzed with the **cobas** test. A high-positive pool (‘Other HR-HPV’) was diluted in HPV-negative master pool to prepare twelve 3-fold serial dilutions. For each dilution, duplicate residual and pelleted samples were tested on the **cobas** test with and without pretreatment.

### Reproducibility

For reproducibility studies, pools of residual SurePath samples were tested with the **cobas** test with and without pretreatment and in the HC2 test on three separate runs over two days. Low-positive pools were selected based on threshold cycles ≥28 and HC2 RFU/cutoff ratios of 3–100. These pools were then tested on two additional runs. For both tests, three replicates were tested during the second run. During the third run, three replicates were tested for the **cobas** test and two replicates were tested in the HC2 test due to pool volume limitations. Samples were stored at room temperature for the duration of the testing.

### Stability

**Fig 1:**
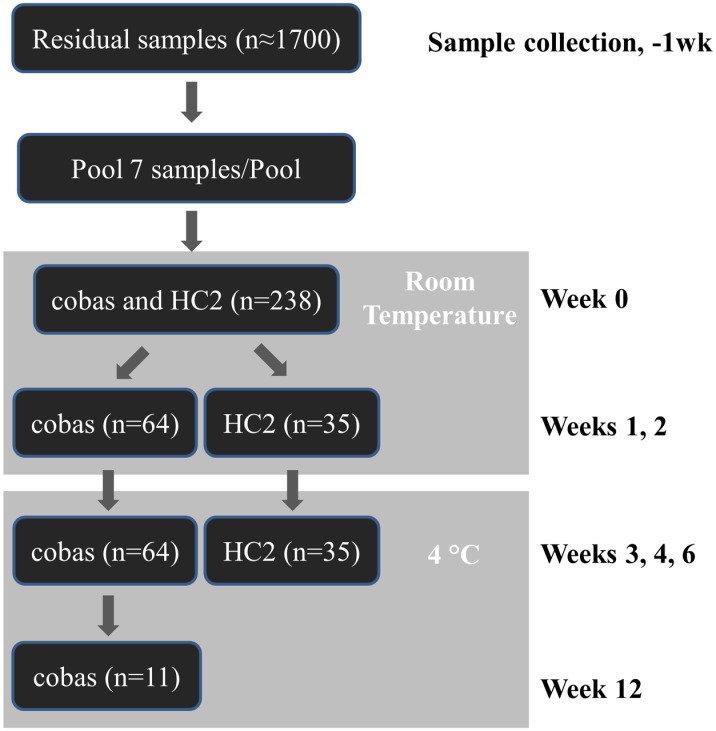
Stability study workflow.

Sample pools were prepared weekly and tested in the **cobas** test with and without pretreatment and in the HC2 test (Fig 1). Pools were selected using threshold cycle and RFU/cutoff ratios described above when possible. Individual pools were tested with either **cobas** or HC2, due to volume constraints and differences in test sensitivities. Pools were stored at room temperature for two weeks (simulating common storage conditions in cytology laboratories) followed by refrigerated storage for an additional 4–10 weeks (simulating common storage prior to HPV testing) [21]. Prior to removing aliquots for testing, pools were vortexed thoroughly. Thirty-five pools were tested by HC2 at weeks 1, 2, 3, and 6; at week 4 only 28 of these pools were tested and none were tested at week 12 due to limited sample volumes. Sixty-four pools were tested by **cobas** at weeks 1, 2, 3, 4, and 6; eleven of these pools were also tested at 12 weeks. Positive results from any channel (Other HR-HPV, HPV16, and HPV18) were analyzed independently, regardless of co-infection.

## Results

### Agreement of cobas and HC2 with sample pools

Without pretreatment, **cobas** and HC2 results agreed for 479 of 682 sample pools (70.2%); 158 pools (23.2%) were **cobas**-positive/HC2-negative, 3 pools (0.4%) were **cobas**-negative/HC2-positive, and 42 pools (6.2%) were equivocal by HC2 (41 **cobas**-positive, 1 **cobas**-negative, Table 1). With pre-analytical treatment, **cobas** and HC2 results agreed for 226 of 315 samples (71.7%); 73 pools (23.2%) were **cobas**-positive/ HC2-negative and 16 pools (5.1%) were **cobas**-positive/HC2-equivocal. Thus, while the positivity rate was greater for **cobas** than for HC2, pretreatment did not change the pattern of agreement.

**Table 1:** Agreement of cobas and HC2 results for pooled cervical cytology samples in SurePath preservative with and without pre-treatment.

**Without pre-treatment**				
	**HC2**			
**cobas**	Positive	Equivocal	Negative	Total
Positive	120 (17.5%)	41 (6.0%)	158 (23.2%)	319 (46.8%)
Negative	3 (0.4%)	1 (0.1%)	359 (52.6%)	363 (53.2%)
Total	123 (18.0%)	42 (6.2%)	517 (75.8%)	682 (100%)
**With pre-treatment**				
Positive	57 (18.1%)	16 (5.1%)	73 (23.2%)	146 (46.3%)
Negative			169 (53.7%)	169 (53.7%)
Total	57 (18.1%)	16 (5.1%)	242 (76.8%)	315 (100%)

### Analytical Sensitivity

#### Cobas vs. HC2

**Fig 2:**
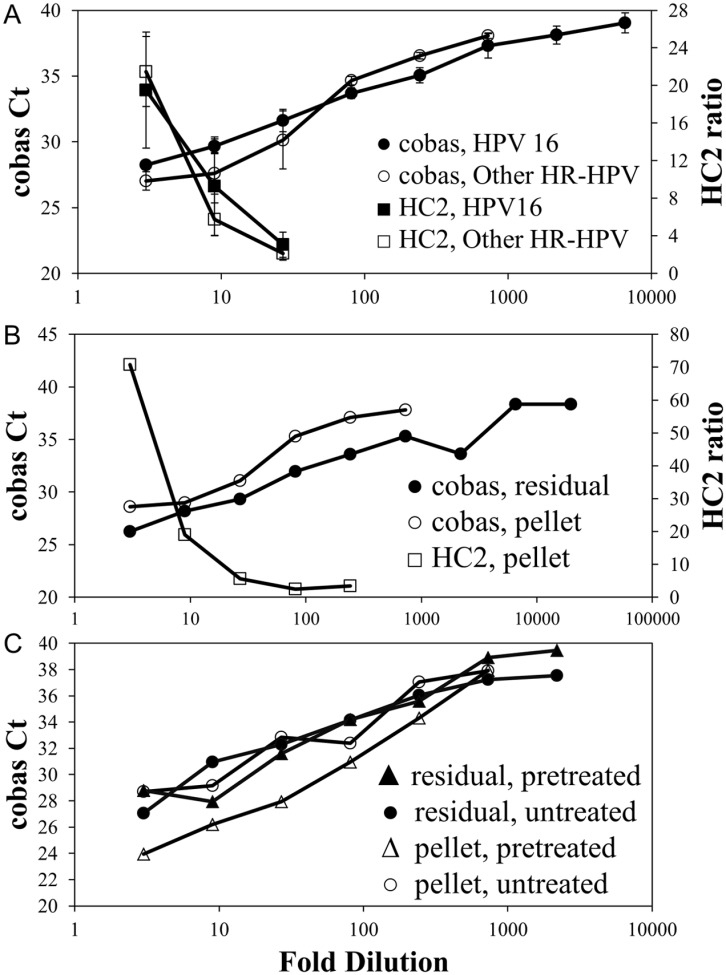
Analytical sensitivity of cobas and HC2 using residual, residual vs. pelleted, and pretreated vs. non-pretreated samples. (A) Serial dilutions of a HPV16-positive and an ‘Other HR-HPV’-positive sample pool showed greater sensitivity for **cobas** than HC2; mean and standard deviation are shown. (B) Serial dilutions of a HPV16-positive sample pool tested as residual sample (no cytology processing, tested by **cobas**) and pelleted sample (after cytology processing, tested by **cobas** and HC2) showed a greater difference in analytical sensitivity with the residual than the pelleted aliquots. Residual aliquots were run in duplicate and the average result is shown; only one replicate was tested for all other dilutions due to limitations in sample volume. (C) Serial dilutions of an ‘Other HR-HPV’-positive residual and pelleted sample pool tested by **cobas** with and without pretreatment showed comparable analytical sensitivity. Each dilution was tested with at least two replicates and average results are shown.

Prior to diluting, both HPV-positive pools had threshold cycle [22] values of 25.4 on the **cobas** test. In serial 3-fold dilutions, 6,561-fold (HPV16) and 729-fold (‘Other HR-HPV’) dilutions were repeatedly detectable on the **cobas** test (Fig 2a) while HC2 was positive up to a 27-fold dilution for both pools. Therefore, the **cobas** test was able to detect HPV16 and ‘Other HR-HPV’ after 27-fold to 243-fold higher dilutions than the HC2 test.

#### Pellet vs. residual sample

To compare analytical sensitivity of pelleted with residual samples, a HPV16-positive pool (original Ct 24.9) was serially diluted, 8 mL of each dilution processed by standard cytology methods, and both pellet (**cobas**, HC2) and residual sample (**cobas**) tested (Fig 2b). A 3-fold difference in the limit of detection was observed for pelleted samples, while **cobas** was still positive with residual sample after 81-fold higher dilution than HC2.

#### Pretreatment vs. no pretreatment

To compare analytical sensitivity of **cobas** with and without pretreatment, serial dilutions of residual and pelleted pools (n = 12) were used. No change in analytical sensitivity was observed with or without pretreatment in residual (2187-fold dilution) or pelleted pools (729-fold dilution, Fig 2c).

### Reproducibility

#### HC2

**Fig 3:**
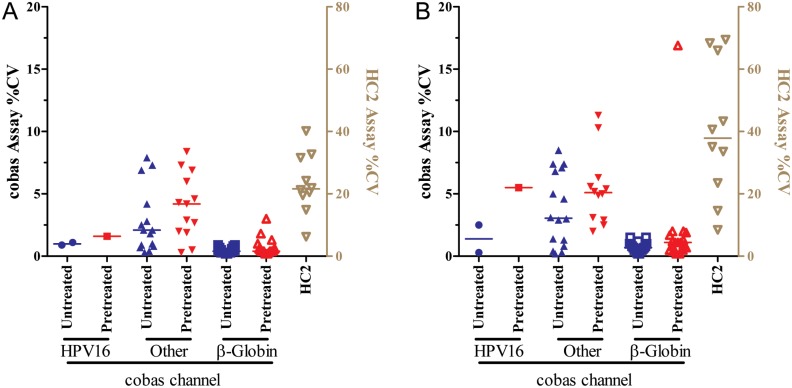
Reproducibility of cobas and HC2 testing. (A) Percent coefficient of variation (%CV) for **cobas** Ct’s (HPV16, ‘Other HR-HPV’, and β-globin) with and without pretreatment and HC2 ratios show median %CV of 1.6 and 1 (**cobas**, HPV16), 4.2 and 2.1 (**cobas**, ‘Other HR-HPV’), 0.4 and 0.4 (**cobas**, β-globin), and 21.6 (HC2 ratio). (B) Inter-run reproducibility was higher with median %CV of 5.5 and 1.4 (cobas, HPV16), 5.1 and 3.05 (**cobas**, ‘Other HR-HPV’), 1.1 and 0.7 (**cobas**, β-globin), and 37.9 (HC2 ratio).

Reproducibility studies using sample pools with RFU/cutoff ratios of 3–84 showed average %CVs of 23% (intra-run) and 40% (inter-run) with the HC2 test (Fig 3a).

#### Cobas, without pretreatment

Sample pools had Ct values between 28.3 and 39.8. The average %CV ranged between 0.5 and 3% for intra-run reproducibility (Fig 3a) and between 0.8 and 4% for inter-run reproducibility (Fig 3b). The %CV correlated significantly with the Ct (p<0.03; r^2^>0.30) in the ‘Other HR-HPV’ channel (S1 Fig).

#### Cobas, with pretreatment

**Fig 4:**
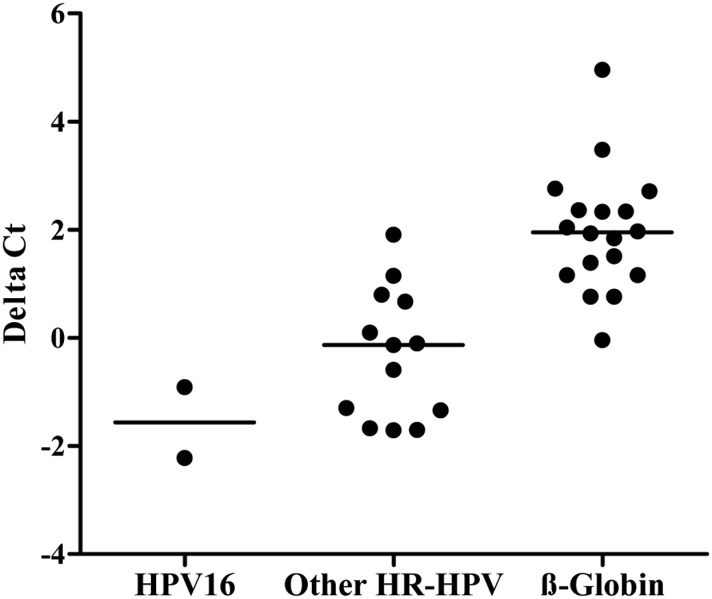
Difference in cobas test Ct’s with and without pretreatment. Delta Ct’s were calculated as Ct with minus Ct without treatment at each timepoint. Median delta Ct was -1.57 for HPV16, -0.13 for ‘Other HR-HPV’, and 1.95 for β-globin (3 runs, ≤7 replicates).

Pretreatment did significantly change %CV for intra-run or inter-run reproducibility. The %CV correlated significantly with the Ct (p<0.03; r^2^>0.30) in the β-globin channel (S1 Fig). Average **cobas** Ct in the β-globin channel was 2 cycles lower with pretreatment than without pretreatment (Fig 4).

### Stability

We used a decline in HC2 ratio and increase in **cobas** Ct as a surrogate for qualitative result changes over the storage period. Even though qualitative result changes are the ideal endpoint for stability studies of HPV tests, the sample sizes required to include sufficient informative (i.e. low-positive) samples is difficult to achieve and results with high-positive samples are of limited utility.

#### HC2

**Fig 5:**
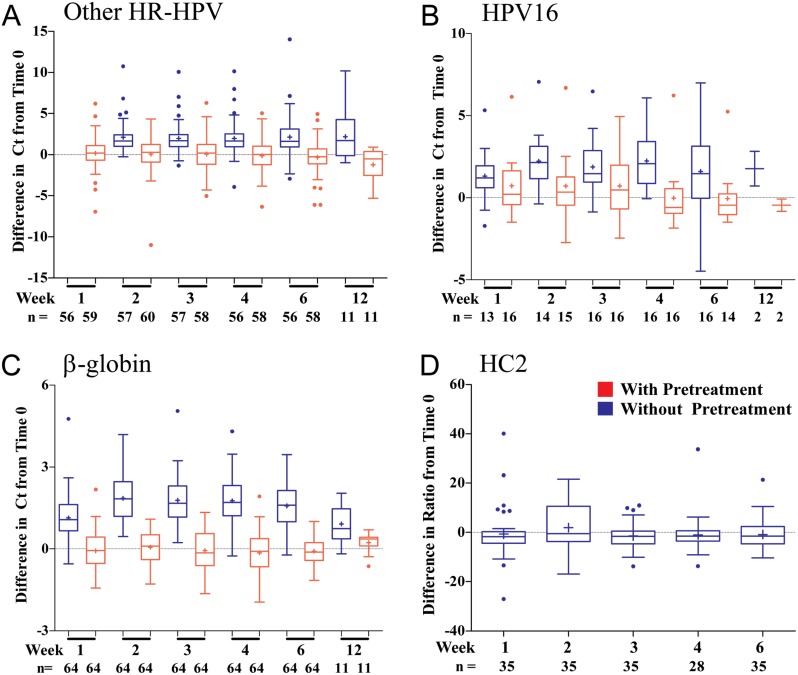
Change in cobas Ct and HC2 ratio over 12 weeks. **Cobas** stability for samples with and without pretreatment for the (A) ‘Other HR-HPV’, (B) HPV16, (C) and β-globin control channels, as well as (D) HC2 results are shown. Differences were calculated by subtracting values at baseline from values at each of the time point of the stability study (whiskers are defined by Tukey; boxes show the interquartile range; mean is indicated by +; dots represent outliers). The number of positive sample pools (n) at each time/treatment point is shown.

At time 0, RFU/cutoff ratios for HC2 ranged from 2.6–30.3. Stability was assessed by comparing ratios at each time point to ratios at time 0 (Fig 5d). The mean differences (+, Fig 5) from baseline were -0.7 (SD 10.7), 2.0 (SD 9.4), -1.5 (SD 5.5), -1.0 (SD 8.0), and -0.9 (SD 6.3) for weeks 1, 2, 3, 4, and 6, respectively. Due to the high variability, differences did not reach statistical significance using the available numbers of pools.

#### Cobas

Of 64 **cobas** HPV-positive pools at time 0, 58 pools were positive for ‘Other HR-HPV’, 17 pools were positive for HPV16 (12 dual positive with ‘Other HR-HPV), and 3 pools were positive for HPV18 (2 dual positive with ‘Other HR-HPV). One pool was positive only for HPV18 and 5 pools were positive only for HPV16. Threshold cycles at time 0 ranged from 26.8–39.9 and 24.7–41.7 (‘Other HR-HPV’), 26.5–39.4 and 26.7–41.7 (HPV16), 33.9–35.1 and 32.4–33.1 (HPV18, data not shown), 27.6–34.1 and 25.8–30.6 (beta-globin) for untreated and pretreated samples, respectively. Without pretreatment, mean differences were 1.6 (week 1, SD 1.9), 2.1 (week 2, SD 1.8), 2.0 (week 3, SD 1.8), 2.0 (week 4, SD 2.1), 2.1 (week 6, SD 2.5), and 2.2 (week 12, SD 3.2). With pretreatment, mean differences were 0.2 (week 1, SD 1.9), 0.1 (week 2, SD 2.2), 0.1 (week 3, SD 2.1), -0.1 (week 4, SD 2.0), -0.3 (week 6, SD 2.0), and -1.2 (week 12, SD 2.0). Only a few pools HPV-positive at time 0 were negative at 6 weeks (two ‘Other HR-HPV’ positive pools with pretreatment, one HPV16 positive with pretreatment, and three HPV16 positive pools without pretreatment). No samples tested at 12 weeks were HPV-negative. There was a significant difference in mean threshold cycles between pretreated and not pretreated samples in the ‘Other HR-HPV’ and β-globin channels (p<0.0001).

## Discussions

The aim of this study was to test stability of cervical cytology samples in SurePath preservative for HR HPV detection with the **cobas** and HC2 tests for up to 12 weeks. We used pooled residual SurePath samples as soon as possible after collection (within one week) as this provides the most realistic estimate of fixation effects in actual patient samples. To simulate common workflows, pooled samples were initially stored at room temperature followed by refrigeration for extended storage.

To prepare sample pools positive for HPV16, HPV18 and the other high-risk HPV types at relevant levels of positivity, 997 sample pools were tested with the **cobas** and HC2 tests. Initial pool testing revealed overall agreement of only 70% for both pretreated and untreated samples with most discordant results (78–82%) being **cobas**-positive/ HC2-negative (Table 1). Given an average positivity rate of ~10% for HR HPV testing in a screening population, the vast majority of pools will have had only one positive sample in the pool thus resulting in a 5 to 7-fold dilution of the positive sample. As **cobas** has greater analytical sensitivity than HC2 [23–25], **cobas**-positive/HC2-negative results are expected in these diluted pools. Consistent with this expectation, the average **cobas** threshold cycle in **cobas**-positive/HC2-positive samples (29.7) was 6.4 cycles lower than in **cobas**-positive/HC2-negative sample pools (36.1, p<0.29). However, the proportion of **cobas**-positive/HC2-negative results (23%) was greater than expected.

To compare analytical sensitivity of **cobas** and HC2 with pooled SurePath samples, we prepared serial 3-fold dilutions of residual HR HPV-positive samples in pools of negative residual samples. Consistent with differences in positivity rates during initial testing of pooled samples, **cobas** was 27 to 243-fold more sensitive than HC2 using residual samples and 3-fold more sensitive using pelleted samples. For these experiments, SurePath samples were used within 7 days of collection to limit formalin fixation effects. However, even within this limited time, crosslinking may have affected analytical sensitivity for HC2 more than for **cobas** when using the same (residual) samples. When using pellets post-cytology processing, the analytical sensitivities of **cobas** and HC2 were comparable. Pretreatment did not affect analytical sensitivity of the **cobas** test. As **cobas** and HC2 have similar clinical sensitivities (>91%) when samples were collected in ThinPrep media [26], the observed differences with SurePath preservative are most likely related to formalin fixation. To compare the clinical sensitivity and specificity of **cobas** and HC2 with samples in SurePath preservative, results need to be compared to clinical endpoints (CIN2+ or CIN3+). This requires large prospective studies. Thus, determining the clinical sensitivity of **cobas** and HC2 with SurePath was outside the scope of this study.

For stability studies, sample pools were chosen based on their **cobas** Cts (26–40) or HC2 ratios (2–31) close to the test cutoffs to increase the likelihood of observing significant effects. This is a departure from pools used in a previous **cobas** study measuring SurePath pool stability where samples were taken from a colposcopy referral population [19]. Those pools are likely to have a higher HPV concentration and therefore, may be less susceptible to noticeable degradation. The SurePath pools used for our study are better suited to assess sample stability since the pools have lower virus concentration and are closer to the test cutoffs. A significant change in mean **cobas** Ct was found between samples with and without pretreatment when the difference in each time point was taken from the baseline time point (time 0) for each pool. The fact that the PCR amplicon for the β-globin control is longer than the PCR amplicons for detection of HPV16 and ‘Other HR-HPV’ is consistent with the observation that the greatest effects of pretreatment were observed for the β-globin control. As pretreatment did not have negative effects on **cobas** sensitivity, these results indicate preferable performance of the **cobas** HPV test when using pretreatment for analysis of samples collected in SurePath preservative. As the pretreatment step is manual and requires generating a sample aliquot, it complicates workflow and has the potential to introduce sample identity errors.

For HC2 stability, 35 SurePath pools were tested over 6 weeks. No significant change in stability was uncovered in the mean difference in HC2 ratios because of the greater variability of this test and the limited number of pooled samples used in this study. A power analysis was prepared based on the detectable difference expected in samples with the HC2 test. This analysis demonstrated that 238 to 943 pools would have to be tested for 80% power to detect significant differences in HC2 ratios over time. This higher number of pools was beyond the scope of this study.

In summary, our study shows that cervical specimen collected in SurePath media are stable for up to 12 weeks for **cobas** testing when using the pretreatment step. Apparent differences in analytical sensitivity of both tests emphasize the need for large clinical performance studies. Unfortunately, these studies are outside the scope of most diagnostic laboratories.

## Supporting Information

S1 Fig %CV for intra-run and inter-run reproducibility. The %CV for intra-run and inter-run reproducibility correlated with the **cobas** Ct’s (*n* = 18) for the β-globin control but not the HPV channels or HC2 ratios (*n* = 10).
(DOCX)
