# Supplementary material for: Cervical Cytology Specimen Stability in Surepath Preservative and Analytical Sensitivity for HPV Testing with the cobas and Hybrid Capture 2 Tests
Source: PLoS One. 2016 Feb 23;11(2):e0149611. doi: 10.1371/journal.pone.0149611 (PMC4764333; doi:10.1371/journal.pone.0149611)
Supplement: S1 Fig — The %CV for intra-run and inter-run reproducibility correlated with the cobas Ct’s (n = 18) for the β-globin control but not the HPV channels or HC2 ratios (n = 10). (DOCX) [file pone.0149611.s001.docx]

**SUPPORTING INFORMATION**

**Supplementary Figure S1**. The %CV for intra-run and inter-run reproducibility correlated with the **cobas** Ct’s (*n*=18) for the β-globin control but not the HPV channels or HC2 ratios (*n*=10). Average %CV for intra-run reproducibility of **cobas** (B) and HC2 (A) testing was determined based on ≤3 replicates. Average %CV for inter-run reproducibility of **cobas** (D) and HC2 (C) testing was determined based on ≤7 replicates over 3 runs.
